# SKN‐1/NRF2 upregulation by vitamin A is conserved from nematodes to mammals and is critical for lifespan extension in *Caenorhabditis elegans*

**DOI:** 10.1111/acel.14064

**Published:** 2023-12-15

**Authors:** Chaweewan Sirakawin, Dongfa Lin, Ziyue Zhou, Xiaoxin Wang, Rhianne Kelleher, Shangyuan Huang, Weimiao Long, Andre Pires‐daSilva, Yu Liu, Jingjing Wang, Ilya A. Vinnikov

**Affiliations:** ^1^ Laboratory of Molecular Neurobiology, Sheng Yushou Center of Cell Biology and Immunology, Department of Genetics and Developmental Biology, School of Life Sciences and Biotechnology Shanghai Jiao Tong University Shanghai China; ^2^ Key Laboratory for Molecular Enzymology and Engineering, School of Life Sciences Jilin University Changchun China; ^3^ Shanghai Key Laboratory of Pancreatic Diseases, Institute of Translational Medicine, Shanghai General Hospital Shanghai Jiao Tong University School of Medicine Shanghai China; ^4^ University of Warwick School of Life Sciences Coventry UK

**Keywords:** aging, *C. elegans*, SKN‐1, vitamin A

## Abstract

Vitamin A (VA) is a micronutrient essential for the physiology of many organisms, but its role in longevity and age‐related diseases remains unclear. In this work, we used *Caenorhabditis elegans* to study the impact of various bioactive compounds on lifespan. We demonstrate that VA extends lifespan and reduces lipofuscin and fat accumulation while increasing resistance to heat and oxidative stress. This resistance can be attributed to high levels of detoxifying enzymes called glutathione S‐transferases, induced by the transcription factor skinhead‐1 (SKN‐1). Notably, VA upregulated the transcript levels of *skn‐1* or its mammalian ortholog *NRF2* in both *C. elegans*, human cells, and liver tissues of mice. Moreover, the loss‐of‐function genetic models demonstrated a critical involvement of the SKN‐1 pathway in longevity extension by VA. Our study thus provides novel insights into the molecular mechanism of anti‐aging and anti‐oxidative effects of VA, suggesting that this micronutrient could be used for the prevention and/or treatment of age‐related disorders.

## Graphical abstract

Vitamin A improves stress resistance and extends longevity in *C. elegans*. SKN‐1 transcription factor is critical for these effects of vitamin A.

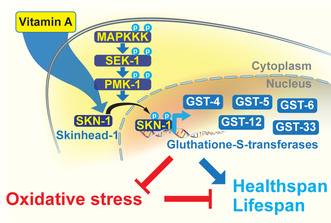

**Abbreviations**
BZIP: Basic leucine zipper type
CCL2: C–C motif chemokine ligand 2
CGC: The Caenorhabditis genetics center
CI: Chemotaxis index
CL2166: *dvIs19[(pAF15)gst‐4p::GFP::NLS] C. elegans* strain
EU1: *skn‐1(zu67)* IV*/nT1[unc‐?(n754)let‐?]*(IV;V) *C. elegans* strain
FDR: False discovery rate
GST: Glutathione S‐transferase
IFN‐β: Interferon‐β
IL‐6: Interleukin‐6
KU25: *pmk‐1(km25)* IV*C. elegans* strain
LB: Lysogeny broth
MAPK: Mitogen‐activated protein kinase
MMP1: Matrix metallopeptidase 1
MS: Mass spectrometry
NFE2: Nuclear factor, erythroid 2
NGM: Nematode growth medium
NRF2 or NFE2L2: Nuclear factor, erythroid 2 like basic leucine zipper type transcription factor 2
ROS: Reactive oxygen species
SASP: Senescence‐associated secretoryphenotype
SEM: Standard error of means
SKN‐1: Skinhead‐1
SS104: *glp‐4(bn2)*I *C. elegans* strain
UPLC: Ultrahigh performance liquid chromatography
VA: Vitamin A

## 1 INTRODUCTION

Gradual aging of the modern human population leads to an increase in age‐related cardiovascular, metabolic, neurodegenerative disorders, and cancer (Niccoli & Partridge, 2012). These diseases constitute a leading cause of morbidity and have a significant impact on both individual quality of life and healthcare costs (Lopreite & Mauro, 2017; Polder et al., 2002). Indeed, longer life in good health brings opportunities to societies as a whole, offering chances to proceed with education and careers. Advances in biological research may provide strategies to prolong the lifespan and increase the health span while delaying the aging process, with nutrient intervention as one of the safe ways to achieve these goals (Kalache et al., 2019). Immoderate dietary habits lead to the development of type 2 diabetes, coronary artery, and cerebrovascular diseases, especially in elder people, whereas reduced dietary intake is linked to malnutrition and poor health status, impaired immune system, muscle weakness, and osteoporosis (Kaur et al., 2019). Thus, maintaining a balanced diet and nutrient intake including essential vitamins and minerals is associated with improved health outcomes and a longer lifespan via reduction in inflammation and maintenance of metabolic homeostasis (Giugliano et al., 2006). Longevity and health span can be directly or indirectly promoted by coenzyme Q10 (Ishii et al., 2004), omega‐3 fatty acids (de Magalhães et al., 2016), resveratrol (Chen et al., 2013), and some vitamins, such as B6 (pyridoxine) (Grootswagers et al., 2021). Indeed, vitamin D (calciferol) improves immune function and reduces the risk of age‐related conditions including heart disease (Lavie et al., 2013), cancer (Weinstein et al., 2015), and cognitive decline (Kang et al., 2021). Vitamin E, an umbrella term for antioxidant and cytoprotective tocopherols and tocotrienols (Dean & Cheeseman, 1987), reduces the risk of chronic diseases related to the accumulation of reactive oxygen species (ROS) (Rizvi et al., 2014). The oxidative stress theory of aging proposes that age‐related damage to cellular functions and tissues is due to an increased accumulation of oxidative damage to the lipids, proteins and DNA by reactive oxygen and nitrogen species (Beckman & Ames, 1998). However, the mechanism by which certain antioxidant vitamins reduce the oxidative damage of the tissue has not been fully understood (Thomas, 2006).

Vitamin A (VA) is a lipophilic group of unsaturated monohydric alcohols with an alicyclic ring. They are represented by retinol, retinyl esters, retinal, retinoic acid, and provitamin A carotenoids (Sommer, 2008). Aside from the critical roles in embryonic development and vision (Ross et al., 2000), VA maintains the immune system by promoting the integrity of intestinal mucus and protecting the oral mucosa (McCullough et al., 1999). VA deficiency may lead to xerophthalmia, night blindness, and impaired immunity in humans, thus affecting the health span and raising the rate of mortality (Diniz Ada & Santos, 2000; Hussain et al., 1995). Mainly affecting low‐income countries, VA deficiency is considered a public health problem. Indeed, children who suffer from VA deficiency are susceptible to infectious diseases (Chen et al., 2009; Qi et al., 2016), diarrhea (Ahmed et al., 2000; Imdad et al., 2010), and measles (D'Souza & D'Souza, 2002), which can lead to premature death (Mayo‐Wilson et al., 2011). Supplementation of developing *Drosophila melanogaster* with increased dietary retinyl palmitate or retinal forms of VA promotes longevity by 17.5% (Massie et al., 1993). Mechanistically, the authors found that VA prevented the superoxide radical‐induced peroxidation of linolenic acid and reduction in cytochrome C. However, the precise mechanism of such protection and the specific pathways involved remain unexplored.

In this work, we used *Caenorhabditis elegans* as a model organism to explore the longevity‐modulating effects of various bioactive compounds including six vitamins. Intriguingly, we found that a VA‐enriched diet significantly extends the healthy lifespan via the upregulation of the SKN‐1 transcription factor. Our study provides new insights into the role of VA in healthy aging and stress resistance.

## 2 RESULTS

**FIGURE 1:**
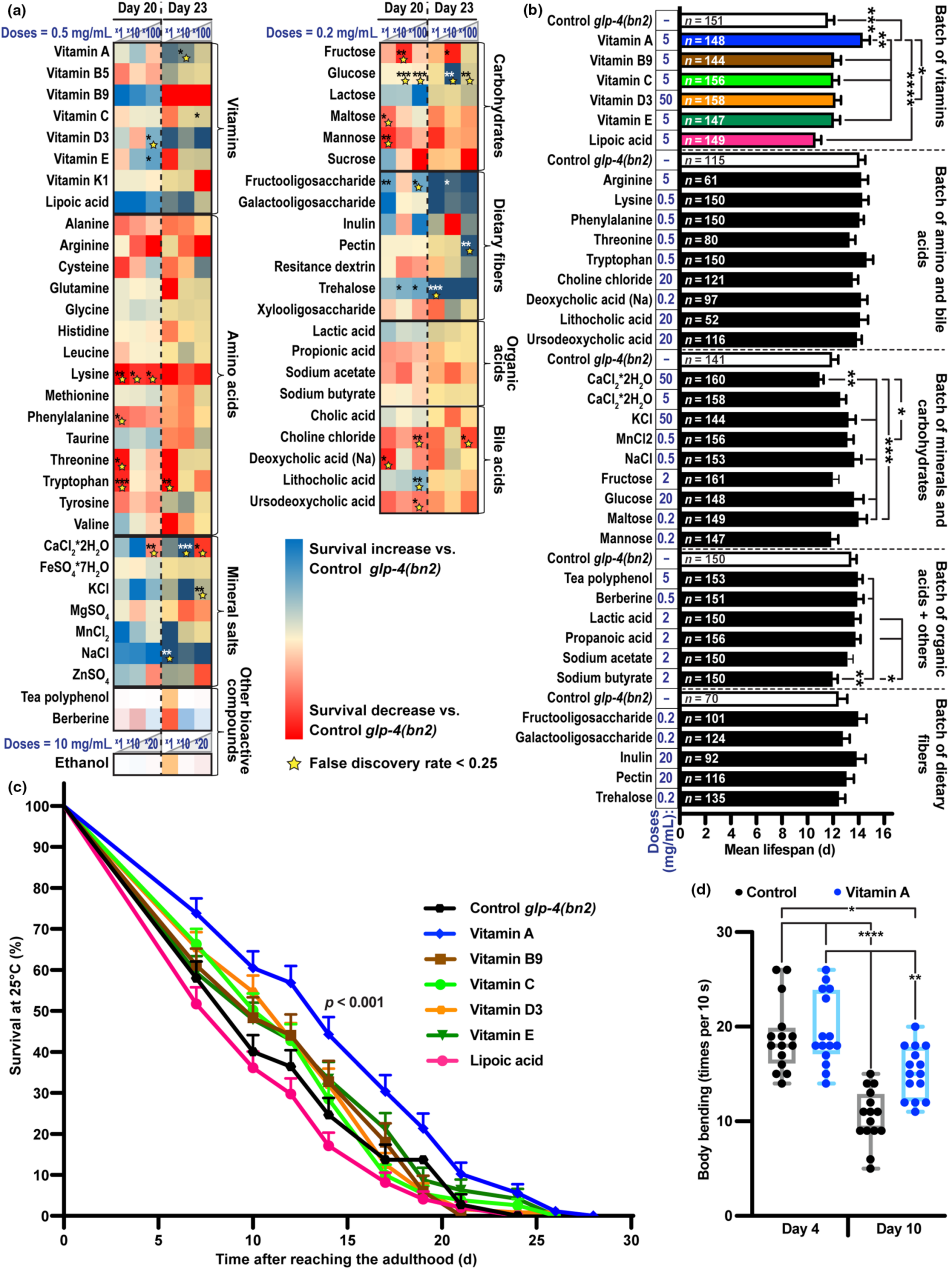
Vitamin A extends lifespan in *Caenorhabditis elegans*. (a) *C. elegans* survival screening of 55 dietary nutrients with indicated dosages at 25°C in a 96‐well plate format (*n* = 50–70 worms per group). Survival differences compared to non‐treated worms on Days 20 and 23 of adulthood are presented as a heatmap with indicated significances, as determined by the Mann–Whitney test with false discovery rate (FDR) correction. Bioactive compounds with FDR <0.25 are labelled with yellow stars. (b, c) Mean lifespan for indicated doses of compounds (b) and survival analysis of the batch with vitamins (c) in a high‐fidelity validation screening with a 6‐cm dish format (significance in [c] is indicated for vitamin A [VA]‐treated vs. control groups, average lifespan extension for VA vs. Control = 23.45%). Other screening data are presented in Tables S4 and S5. (d) Body bending assessed on Days 4 and 10 of adulthood in wild‐type *C. elegans* treated with 5 mg/mL vitamin A (*n* = 15). Data are presented as mean ± SEM. *p < 0.05; **p < 0.01; ***p < 0.001; ****p < 0.0001. OASIS2 followed by unpaired one‐way ANOVA and Tukey's post hoc test (b) and log‐rank test (c) one‐way ANOVA followed by Tukey's post hoc test (d) were used for other statistical analyses.

In order to investigate the impact of 55 dietary nutrients intake on survival, we performed a 96‐well plate format primary screening with up to three wide‐range doses of these bioactive compounds in *C. elegans* grown on Nematode Growth Medium (NGM) and OP50 *E. coli* as their food source (Figure 1a, Tables S1–S3). Strikingly, out of 34 compounds selected for the high‐fidelity validation screening on 6‐cm dishes, only VA significantly affected longevity, with the mean lifespan extended by this vitamin by 23.45% (Tables S4 and S5, Figure 1b,c). Moreover, VA attenuated aging‐associated decline in motility (Figure 1d). Our data thus confirm the VA‐mediated lifespan extension in invertebrates, which has been previously reported by Massie et al. on *D. melanogaster* (Massie et al., 1993). Notably, despite the overall downward slope of children mortality attributable to VA deficiency‐associated pathological conditions (Figure S1a), its close‐to‐horizontal projection in Sub‐Saharan Africa and South Asia is alarming, to say the least, as also indicated elsewhere (Stevens et al., 2015). Although not related to aging, VA deficiency‐associated mortality analyzed by the authors directly impacts average life expectancy in these populations and hence deserves further studies.

**FIGURE 2:**
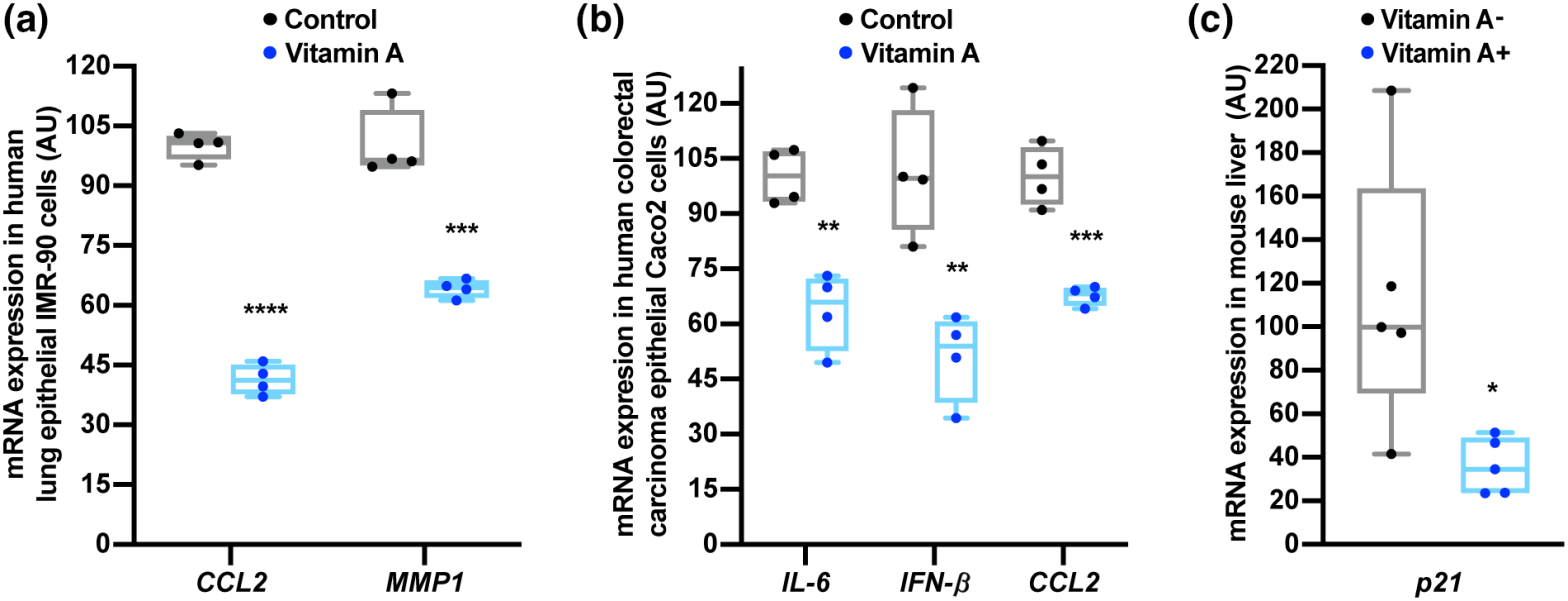
Anti‐inflammatory and anti‐senescence properties of vitamin A in mammals. (a, b) RT‐qPCR analysis of pro‐inflammatory cytokines and senescence‐associated transcripts in human lung epithelial IMR‐90 cells (a) and human colorectal carcinoma epithelial Caco2 cells (b). (c) RT‐qPCR analysis of *p21* transcript in the liver tissues of mice treated for 10 weeks with VA‐deficient or the same diet (AIN‐93G) containing 4000 IU/kg of VA. Data are presented as mean ± SEM. *p < 0.05; **p < 0.01; ***p < 0.001; ****p < 0.0001 as analyzed by Student's unpaired *t* test.

Next, we investigated whether vitamin A is able to regulate aging‐associated markers also in mammals. Interestingly, we detected a significant decline in the transcript levels of senescence‐associated genes and pro‐inflammatory cytokines: C‐C motif chemokine ligand 2 (CCL2), matrix metallopeptidase 1 (MMP1), interleukin‐6 (IL‐6), and interferon‐β (IFN‐β) in human lung epithelial IMR‐90 (Figure 2a, Table S6) or human colorectal carcinoma epithelial Caco2 (Figure 2b, Table S6) cells exposed to VA. Moreover, mice receiving vitamin A‐deficient diet for 10 weeks revealed an increase in senescence‐associated transcript *p21* in the liver tissue, compared to animals fed with the same diet containing recommended levels of vitamin A (4000 IU/kg) (Figure 2c, Table S6). These results suggest that the health span‐ and/or life span‐promoting action of VA is conserved from invertebrates to mammals.

**FIGURE 3:**
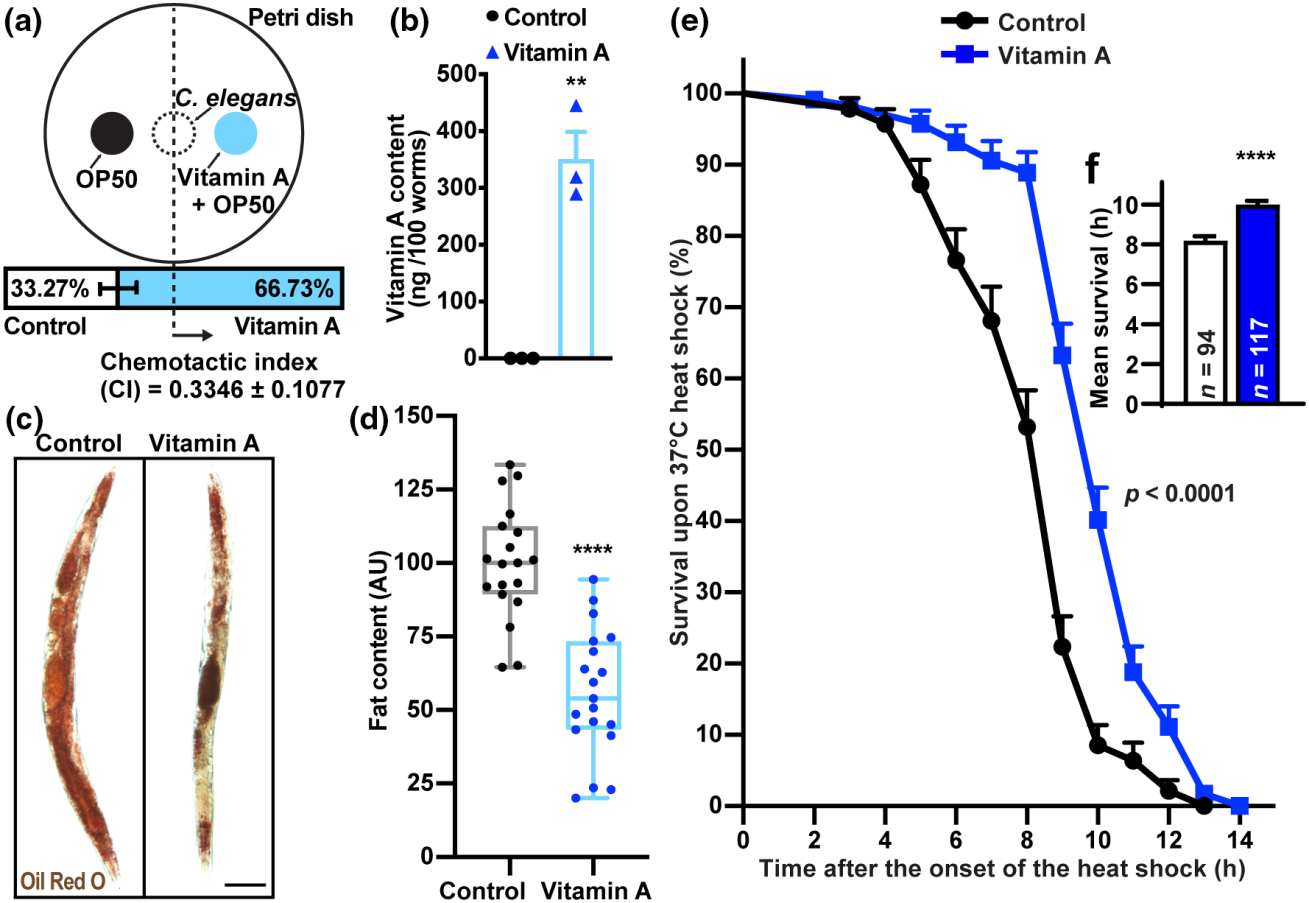
Phenotypic and morphological characterization of vitamin A‐treated *Caenorhabditis elegans*. (a, b) Chemotaxis assay (a, *n* = 136), body concentration of VA (b) in *C. elegans* treated with 5 mg/mL VA. (c, d) Representative microphotographs (c) and quantification (d) of fat content assessed by Oil Red O staining in wild‐type worms treated with 5 mg/mL VA for 5 days (*n* = 19). (e, f) Survival analysis (e) and mean lifespan (f) of *C. elegans* treated with 5 mg/mL VA under 37° heat stress (average survival extension = 22.12%). All experiments were repeated three times. Data are presented as mean ± SEM. **p < 0.01; ****p < 0.0001 as analyzed by Student's unpaired *t* test. Scale bar (in μM): 100.

Since metabolic imbalance‐associated pathologies are among the leading causes of low life expectancy and early mortality (Khan et al., 2018; Peeters et al., 2003), we next sought to exclude the VA‐mediated food aversion as a reason of food intake‐restricted longevity promotion (Piper & Bartke, 2008) in *C. elegans*. On the contrary, *C. elegans* demonstrated a positive chemotaxis to VA (Figure 3a), thus ruling out the abovementioned mechanism of lifespan extension. Removal of germ cells (Arantes‐Oliveira et al., 2002), other changes in growth, development and locomotion, and energy intake decline (Piper & Bartke, 2008) can lead to a dramatic increase in the lifespan of *C. elegans* (Huang et al., 2004). Thus, we next tested these parameters in worms treated with VA. While observing markedly upregulated VA levels in the tissues of nematodes (Figure 3b), we detected no change in fecundity (Figure S1b), body length and width (Figure S1c,d), no decline in locomotion (Figure 1d, Figure S1e), or signs of imbalanced energy homeostasis such as food intake (assessed by pharyngeal pumping rates [Rodríguez‐Palero et al., 2018]) in VA‐exposed *C. elegans* (Figure S1f). Despite the lack of hypophagia (Figure S1f), we sought to determine the levels of fat accumulation in *C. elegans* treated with VA. Intriguingly, VA significantly reduced the whole‐body lipid deposition in *C. elegans* (Figure 3c,d), which did not hinder them to cope with the heat stress better than the control animals (Figure 3e,f). Such enhancement of health span in VA‐treated nematodes might be related to a decreased oxidation of lipids and proteins, a well‐known marker associated with aging (Holmstrup et al., 2010; Slimen et al., 2014).

**FIGURE 4:**
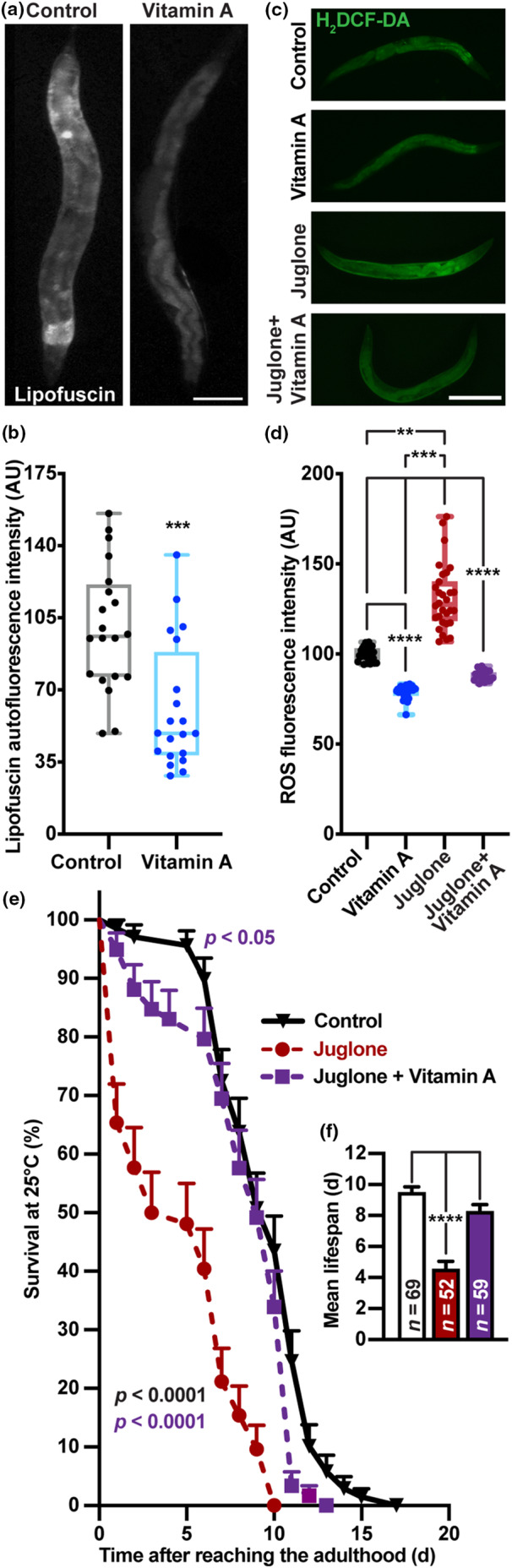
Vitamin A reduces oxidative stress. (a, b) Representative microphotographs (a) and quantification (b) of lipofuscin autofluorescence at excitation/emission 340/430 nm (a, b, *n* = 20) in wild‐type worms treated with 5 mg/mL VA for 5 days. (c, d) Representative microphotographs (c) and quantification (d) of intracellular reactive oxygen species levels in wild‐type worms after 4 days of 5 mg/mL VA treatment with (*n* = 25 and 30 for vehicle + juglone and VA + juglone groups, respectively) or without (*n* = 25) 240 μM juglone exposure. (e, f) Survival assay (e) and mean lifespan (f) in wild‐type worms treated with 240 μM juglone and 5 mg/mL VA (average survival change vs. Control = −51.69% and −12.55% for Juglone and Juglone + VA groups, respectively). Experiments were repeated three times. Data are presented as mean ± SEM. Survival curves were analyzed by log‐rank test, mean lifespans—by OASIS2 and compared by one‐way ANOVA followed by Tukey's post hoc test, two group comparisons—by Student's unpaired *t* test. *p < 0.05; **p < 0.01; ***p < 0.001; ****p < 0.0001 with significances in (e) versus groups indicated by respective colors. Scale bars (in μM): 200 (a), 320 (c, e).

Indeed, treatment of worms with VA reduced the levels of lipofuscin, a pigment composed of highly oxidized proteins and lipids (Kennedy et al., 1995). They accumulate in intestinal cells (Clokey & Jacobson, 1986) in response to oxidative damage and aging (Terman & Brunk, 1998, 2006) (Figure 4a,b). These results suggest that VA improves the health span and life span of *C. elegans* via inhibition of lipid accumulation and oxidation. Accordingly, we detected a significant decline in the whole‐body ROS accumulation after VA pre‐treatment with or without the exposure to juglone, a potent oxidant (Figure 4c,d), suggesting that this reduction in oxidative damage to macromolecules such as lipids, DNA, and proteins may slow down the aging (Beckman & Ames, 1998). Strikingly, in addition to normalization of ROS levels upon juglone exposure (Figure 4c,d), treatment by VA also rescued survival in worms exposed to this oxidant (Figure 4e,f). These results indicate that VA enhances tolerance against oxidative stress thus improving the health span in nematodes.

**FIGURE 5:**
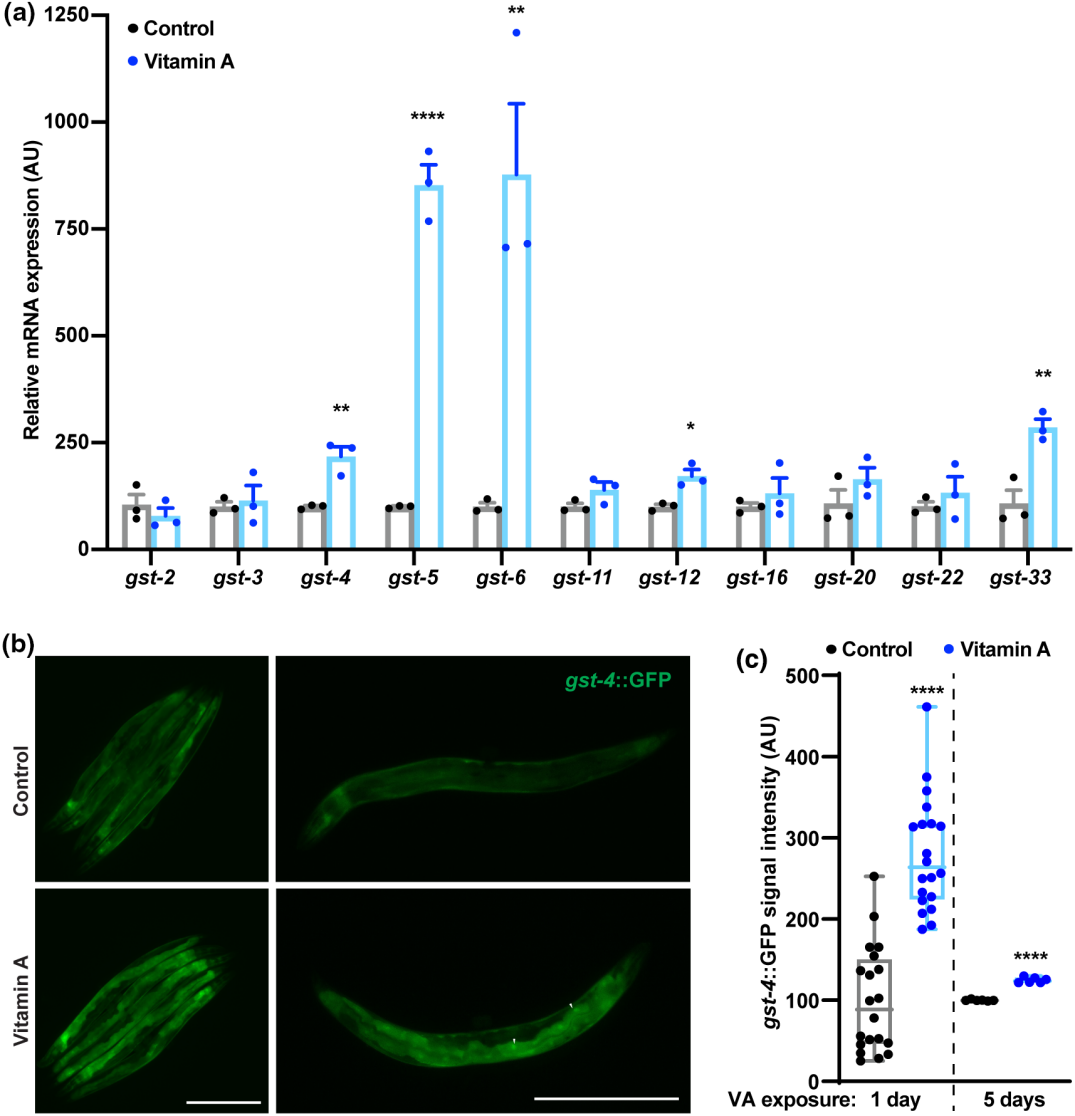
Vitamin A stimulates expression of glutathione S‐transferases in *Caenorhabditis elegans*. (a) Expression of glutathione S‐transferase (*gst*) genes in *C. elegans* treated with 5 mg/mL VA (*n* = 3). (b, c) Representative microphotographs of *gst‐4* promoter‐driven GFP in CL2166 (lsgst‐4p::GFP) worms after 1 day of incubation with 5 mg/mL VA (b) and quantification for 1‐day (*n* = 20) and 5‐day (*n* = 6) incubations (*n* = 20) (c). The experiment was repeated three times. Data were analyzed by Student's unpaired *t* test and presented as mean ± SEM. *p < 0.05; **p < 0.01; ****p < 0.0001. Scale bar (in μM): 640.

Next, we determined the transcription levels of several glutathione S‐transferases (GSTs) (Figure 5a), the key enzymes activated in response to and counteracting oxidative stress (Singhal et al., 2015). Interestingly, among the genes tested, VA elevated expression of *gst‐5*, *‐6*, *‐12*, *‐33*, and *‐4* (Figure 5a, Table S6). The latter was also confirmed in the *gst‐4p::GFP* transgenic reporter strain of *C. elegans* after 1 or 5 days exposure to VA (Figure 5b,c). Remarkably, all of these genes can be activated by transcription factor skinhead‐1 (SKN‐1) (Detienne et al., 2016; Oliveira et al., 2009; Park et al., 2009). SKN‐1, a functional equivalent of nuclear factor, erythroid 2 (NFE2) like basic leucine zipper type (BZIP) transcription factor 2 (NRF2 or NFE2L2) in mammals, promotes longevity and innate immunity via upregulation of detoxifying genes in response to oxidative stress (An & Blackwell, 2003; Papp et al., 2012).

**FIGURE 6:**
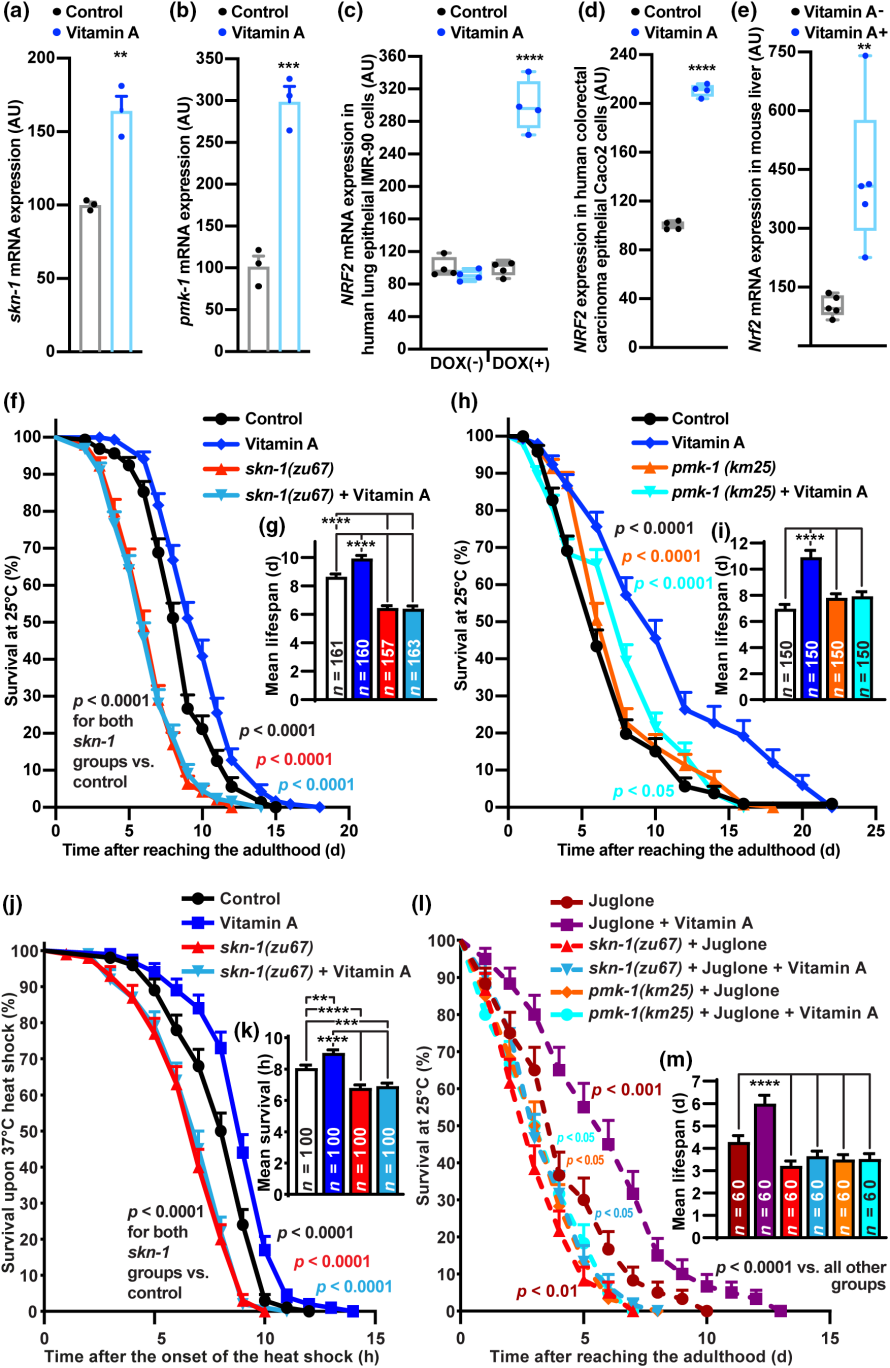
The PMK‐1/SKN‐1 pathway is critical for lifespan extension by vitamin A. (a, b) Expression of *skn‐1* (a) and *pmk‐1* (b) in *Caenorhabditis elegans* treated with 5 mg/mL VA (*n* = 3). (c, d) Expression of *NRF2* transcripts in human lung epithelial IMR‐90 cells after senescence induction by doxorubicin (DOX) (c) and human colorectal carcinoma epithelial Caco2 cells (d) treated with 5 mg/mL VA (*n* = 3). (e) Expression of *Nrf2* in the liver tissues of mice fed for 10 weeks with VA‐deficient diet (*n* = 5). (f–i) Survival analysis (f, h) and mean lifespan (g, i) of *skn‐1(zu67)* (f, g) and *pmk‐1(km25)* mutants (h, i) treated with 5 mg/mL VA. Average survival change versus Control = 14.94%, −25.37%, and −25.84% for VA, *skn‐1(zu67)* and *skn‐1(zu67)* + VA groups, respectively; = 56.44%, 11.89%, and 13.61% for VA, *pmk‐1(km25)* and *pmk‐1(km25)* + VA groups, respectively. (j, k) Survival analysis (j) and mean lifespan (k) of *skn‐1(zu67)* mutants treated with 5 mg/mL VA under 37° heat stress (average survival change vs. Control = 12.02%, −15.73%, and −14.37% for VA, *skn‐1(zu67)* and *skn‐1(zu67)* + VA groups, respectively). (i–m) Survival analysis (l) and mean lifespan (m) of *skn‐1(zu67)* and *pmk‐1(km25)* mutants treated with 5 mg/mL vitamin A with or without 240 μM juglone (average survival change vs. Juglone group = 40.18%, −24.76%, −14.71%, −18.22%, and −17.75% for Juglone‐VA, *skn‐1(zu67)* + juglone, *skn‐1(zu67) +* Juglone + VA, *pmk‐1(km25)* + Juglone, and *pmk‐1(km25) +* Juglone + VA groups, respectively). Data are presented as mean ± SEM. Survival curves were analyzed by log‐rank test, mean lifespans—by OASIS2 and compared by one‐way ANOVA followed by Tukey's post hoc test, two groups comparisons—by Student's unpaired *t* test. **p < 0.01; ***p < 0.001; ****p < 0.0001 with significances in (f, h, j, l) versus groups indicated by respective colors.

Interestingly, VA‐exposed *C. elegans* revealed upregulation of the transcript levels of both *skn‐1* and its upstream activator *pmk‐1* (Figure 6a,b, Table S6), suggesting that the p38 mitogen‐activated protein kinase (MAPK)/SKN‐1 pathway may extend lifespan via protection against oxidative stress. The buildup of ROS during aging can trigger cellular senescence, characterized by a cessation of cellular proliferation in response to oxidative stress (Liguori et al., 2018). SKN‐1 and its mammalian ortholog NRF2 are well‐known transcription factors to counteract the senescence‐associated secretory phenotype (SASP) (Lu et al., 2021; Romero et al., 2019; Zhou et al., 2016). Strikingly, in human lung epithelial IMR‐90 cells, VA drastically upregulated *Nrf2* transcript levels to mitigate the doxorubicin‐induced senescence (Figure 6c, Table S6). Accordingly, we detected elevation of this transcription factor mRNA expression in human colorectal carcinoma epithelial Caco2 cells (Figure 6d, Table S6). Moreover, mice on vitamin A‐deficient diet demonstrated a decrease in *NRF2* transcript levels in the liver tissue, compared to animals fed with the same diet containing recommended levels of vitamin A (4000 IU/kg) (Figure 6e, Table S6).

To prove the causal role of p38 MAPK/SKN‐1 pathway in VA‐mediated longevity extension, we used the loss‐of‐function *skn‐1(zu67)* and *pmk‐1(km25) C. elegans* mutants. Indeed, VA failed to extend the lifespan upon inactivation of two out of four isoforms of *skn‐1* (Figure 6f,g) and attenuated the VA‐mediated lifespan extension upon inactivation of PMK‐1, a kinase that is required for phosphorylation‐dependent translocation of the active form of SKN‐1 to the nucleus (Inoue et al., 2005) (Figure 6h,i). In line with the abovementioned findings, VA failed to extend the survival of *skn‐1(zu67)* worms under heat stress (Figure 6j,k) or lifespan of *skn‐1(zu67)* and *pmk‐1(km25)* mutants exposed to juglone (Figure 6i,m). Our data thus prove that SKN‐1 promotes VA‐mediated lifespan extension via increase in stress resistance.

## 3 DISCUSSION

With gradually increasing life expectancy, modern societies have set an ambitious goal to promote healthy aging. The latter is dependent on many factors, including maintaining a balanced diet. Due to a high degree of evolutionary conservation in pathways related to metabolism and aging (Shen et al., 2018; Wählby et al., 2014), *C. elegans* is a well‐established in vivo model for longevity screening of drugs and bioactive compounds. In this study, we (i) identify the lifespan‐promoting effect of VA in *C. elegans*, (ii) demonstrate that this micronutrient enhances heat stress and oxidative stress resistance, and (iii) prove that the antioxidative SKN‐1 pathway is critical for longevity promotion by VA in *C. elegans*. Moreover, (iv) VA‐dependent upregulation of the SKN‐1 ortholog NRF2 in human cells accompanied by downregulation of pro‐inflammatory and senescence‐associated genes suggests an evolutionary conservation of VA‐mediated protection from nematodes and insects (Massie et al., 1993) to mammals. Indeed, VA‐mediated reduction in pro‐inflammatory SASP factors could lead to a decrease in immune cell infiltration (Finkin et al., 2015; Shaw et al., 2013; Singh et al., 2008) and a slowdown in age‐related functional decline of adaptive immune system, ultimately slowing down the aging process (Freund et al., 2010; Liu et al., 2019). These data might be further supported by reciprocal changes in liver expression levels of *NRF2* and *p21* in mice fed with VA‐deficient diet. Even though the pro‐inflammatory and senescence genes had been widely used as age‐related markers in vitro and in vivo (Bruunsgaard et al., 2003; Gordon et al., 2011; Li et al., 2023), they can be also upregulated in other conditions, not related to aging (Ferrucci & Fabbri, 2018; Vasto et al., 2007). Notably, our analysis of the prevalence of vitamin A deficiency and associated deaths in children (Stevens et al., 2015) highlights the persisting importance of availability of this micronutrient in diets and urges the need for targeted interventions in the Sub‐Saharan Africa and South Asia to prevent mortality caused by VA deficiency‐associated pathological conditions. Although the latter phenomenon is not associated with aging, it is strongly related to longevity in these populations. In this study, we demonstrate that this vitamin is essential for extending healthy longevity in *C. elegans* without any detrimental impact on growth, fecundity, motility, or food intake.

Overeating, resulting in excessive lipid accumulation and obesity, is the strongest modifiable aging‐associated risk factor from *C. elegans* to mammals (Ahima, 2009; Fontaine et al., 2003; Tam et al., 2020; Van Gilst et al., 2005; Wang et al., 2020; Zhang et al., 2015). Indeed, calorie restriction increases the lifespan in *C. elegans* (Wu et al., 2019) and mice (Weindruch et al., 1986), while the high‐fat diet administration leads to higher mortality in mice (Zhang et al., 2015). In *C. elegans*, lipid accumulation leads to lipotoxicity resulting in progression of age‐related pathologies (Palikaras et al., 2017). These effects may be associated with complex interactions between more than 400 genes that are involved in fat metabolism (Shen et al., 2018). Interestingly, accumulation of certain lipids, such as ω‐6 polyunsaturated fatty acids, increases lifespan in *C. elegans* (O'Rourke et al., 2013) whereas muscle loss, weight loss, and malnutrition in the elderly can lead to a shortened life span (Bales & Ritchie, 2002; Newman et al., 2001). Indeed, lipids accumulate large amounts of energy, which can be released on demand and thus help cope with stressor factors and in some conditions contribute to increased health span and longevity. However, for healthy aging, it may be decisive, which type of fat is deposited in the body, with some types being protective and others detrimental, such as saturated fatty acids (Arsenyadis et al., 2022; Berg et al., 2020; Jain et al., 2015; Zhou et al., 2020). In this study, we detected a VA‐dependent decrease in whole‐body fat accumulation, which did not alter the heat stress resistance. The latter is known to decrease with age (Dues et al., 2016; Kenney & Hodgson, 1987) and increase in *C. elegans* mutants with increased longevity (Lithgow et al., 1995). We show that heat stress resistance is also drastically improved upon VA treatment.

Excessive fat deposition may result in increased oxidation of lipids, which is associated with aging (Holmstrup et al., 2010; Slimen et al., 2014).Together with accumulation of ROS, enhanced lipid oxidation may lead to inflammation (Bulua et al., 2011; Mittal et al., 2014) and increase the risk of chronic diseases such as cancer (Finkel & Holbrook, 2000; Reuter et al., 2010). Moreover, accumulation of both aging pigment lipofuscin (Pincus et al., 2016; Terman et al., 2004) and ROS is diminished in *C. elegans* treated with VA demonstrating that this vitamin protects against oxidative stress. There are two major ways to counteract the deleterious effects of ROS overproduction: extrinsic alimentary antioxidants and endogenous antioxidants or detoxifying enzymes (Gilgun‐Sherki et al., 2001). Dietary antioxidant deficiencies may increase the risk of chronic age‐related diseases (Pham‐Huy et al., 2008). Here we show that VA treatment leads to induction of antioxidative enzymes, glutathione‐S‐transferases, that are downstream of the transcription factor SKN‐1. The latter is orthologous to the mammalian Nrf2 protein playing an essential role in oxidative stress defense (Nguyen et al., 2009) by inducing the transcription of multiple detoxifying genes (Das et al., 2013).

Indeed, the protective effect of VA via activation of SKN‐1/Nrf2 has been demonstrated in the neuronal (Zhao et al., 2009) and liver (Wang et al., 2014) tissues. According to our in vitro experiments, VA reduces inflammation and cellular senescence, while upregulating the expression of *NRF2*. Strikingly, this finding was supported by mRNA expression data from liver tissues of mice fed with VA‐deficient food or the same diet containing recommended levels of vitamin A (4000 IU/kg). Moreover, expression of both *skn‐1* and its upstream activator *pmk‐1* was induced by VA in *C. elegans*. In conclusion, this study points to the critical involvement of SKN‐1 in VA‐mediated longevity extension via increase of stress resistance. Our data thus suggest that balanced diets including vitamins, such as VA, play a critical role in health span and longevity.

## 4 EXPERIMENTAL PROCEDURES

### 4.1 Reagents, compounds, and nutrients

With the exception of the primary screening experiment (see Table S1 for the reagents used), all experiments on *C. elegans* were conducted with 5 mg/mL VA in a form of retinol (Zhejiang NHU Co., Ltd, #127‐47‐9), which was resuspended in water (assisted by ultrasound) and distributed (using beads) on the surface of the NGM plate (100 μL per 6‐cm dish) after it had fully cooled down to room temperature after autoclaving (to prevent degradation of VA), but before the addition of *Escherichia coli* strain OP50. For all substances, reconstitution was performed in water to avoid an interference of organic solvents with the physiology of *C. elegans*. Hence, some of the compounds formed not a solution but a suspension that was applied on the surface of the plates followed by drying and application of *E. coli*. Resuspension and all experimental procedures with VA were conducted in dark to avoid light‐dependent degradation.

### 4.2 Animals

The wild‐type *C. elegans* Bristol N2 and transgenic strains were obtained from the Caenorhabditis Genetics Center (CGC). These included *glp‐4(bn2)*I (further referred to as SS104); *pmk‐1(km25)*IV (further referred to as KU25); *dvIs19[(pAF15)gst‐4p::GFP::NLS]* (further referred to as CL2166), and *skn‐1(zu67)*IV/*nT1[unc‐?(n754)let‐?]* (IV;V) (further referred to as EU1). The *Escherichia coli* strain OP50 (CGC) was grown aerobically at 37°C in Luria‐Bertani (LB) broth. Nematodes were maintained at 15°C on a standard NGM with 10‐μM antifungal nystatin (Sangon‐Biotech) and *E. coli* OP50 as a food source. Primary and secondary screening were conducted on the SS104 strain, while all other experiments *C. elegans* were performed on N2 wild‐type worms to avoid any phenotypic changes associated with germline ablation. For the same reason, we have not used FuDR, but transferred the adults to a new plate. Synchronized wild‐type N2 worms were grown on NGM plate until they reached L4 stage and then were transferred to 25°C. On the next day, synchronized Day 1 adult stage worms were transferred to the plates with or without 5 mg/mL VA to perform stress tolerance assay, measurements of fecundity, body size, body bending, pharyngeal pumping rate, lipofuscin, ROS and fat content. For ROS, lipofuscin, and fat content measurements, worms were collected and washed with M9 buffer. For fecundity analysis, three worms per condition were allowed to lay eggs until the end of the reproductive period. Each worm was transferred to new plate every 24 h, and the number of hatched progeny was scored after 48 h. To analyze the body dimensions and lipofuscin accumulation in adult worms 5 days after VA treatment, they were anesthetized with 10 mM levamisole (Aladdin), mounted on the 2% agarose pad and examined and photographed with stereo microscope (Olympus MVX10). Lengths were measured from the nose to the tail widths—from side to side at the position of vulva with ImageJ program. Lipofuscin autofluorescence at the blue range (excitation and emission wavelengths: 340 and 430 nm, respectively) was detected using an ECHO revolve fluorescence inverted integrated microscope (RVL‐100‐G) and quantified by using ImageJ software (the experiments were independently repeated at least three times). To measure bending rates of the adult worms on Days 4 and 10, a drop of M9 buffer was applied on the worms after they have been incubated with VA for 5 days, and the bending during 10 s was filmed using Olympus MVX10 microscope followed by analysis. Likewise, pharyngeal pumping within 10 s was filmed by Olympus MVX10 and analyzed in worms after treatment with VA for 5 days (the experiments were independently repeated at least three times). To measure ROS levels 4 days after VA treatment, worms were collected, washed with M9 buffer, and incubated for 1 h at 20°C with 100 μM 2,7‐dichlorodihydrofluorescein diacetate H_2_DCF‐DA (Beyotime Biotechnology, Ex/Em 470/550 nm), a fluorescent redox probe commonly used for ROS detection (Afri et al., 2004). Worms were anesthetized with 10 mM levamisole (Aladdin) and mounted on the 2% agarose pad. The images are acquired using ECHO revolve fluorescence microscope (ECHO RVL‐100‐G) and the fluorescence intensity was quantified using ImageJ software (the experiments were independently repeated at least three times). Similarly, GFP expression in the CL2166‐GFP reporter line was detected and quantified 1 day and 5 days after incubation with VA. Thermal tolerance assay was performed 5 days of VA treatment by applying 37°C heat shock. Survival was scored every hour till death by touch‐provoking method (the experiments were independently repeated at least three times). For fat storage measurements in *C. elegans* treated with VA for 5 days, they were collected and washed with M9 buffer, permeabilized with MRWB buffer and 2% Triton X‐100 (Sigma) followed by staining with 1% Oil Red O (Sigma) solution, washing with 60% isopropanol and M9 buffer. Worms were mounted on 2% agarose pad and imaged with Olympus MVX10 microscope. Data were analyzed using ImageJ software as previously reported (Schneider et al., 2012) (the experiments were independently repeated at least three times).

All experimental procedures on mice were conducted at the animal facility of Shanghai Jiao Tong University, Shanghai, China in accordance with institutional and international standards and approved by the local authorities. Ten male C57BL/6 mice obtained from SLAC Inc. were housed (five mice/cage) in IVC system cages (Dimensions 325 × 210 × 180 mm; Suzhou Fengshi Laboratory Animal Equipment Co., Ltd) with a 12 h light/dark phase cycle at 22.0 ± 1°C with ad libitum access to water and food. To limit the interactions between microbiome and vitamin A metabolism, the 32‐week‐old mice were pre‐treated with an antibiotic cocktail containing ampicillin (1 g/L), vancomycin (0.5 g/L), metronidazole (1 g/L), and neomycin (1 g/L) in drinking water for 7 weeks, then were fed for 10 weeks with VA‐deficient or the same diet (AIN‐93G) containing recommended levels of VA (4000 IU/kg of VA, 110700, Dyets). At the end of the experiment, mice were sacrificed during the light phase in a fed state and their livers were snap‐frozen in liquid nitrogen for subsequent RNA isolation and qPCR analysis.

### 4.3 Lifespan assays

All lifespan assays were conducted at 25°C unless otherwise mentioned. Worms were synchronized using bleaching technique as described before (Bar‐Ziv et al., 2020). Synchronized eggs were allowed to grow on NGM plate at 15°C until they reached early four‐stage larvae (L4), which were then shifted to 25°C. On the next day, young‐adult stage worms were cultured on the plates that have been coated with different compounds and overnight culture of fresh *E. coli* OP50. Adult stage worms were transferred to new plates every day until the end of the egg‐laying period. In the primary screening (Figure 1a, Tables S2 and S3), we cultured young adult‐stage worms (from day 0 of the adulthood) on NGM‐covered 96‐well plates that were seeded with *E.coli* OP50 at 25°C. Worms were scored as dead or as alive by touch‐provoked method with a platinum wire. See Tables S2–S5 and the main figures for detailed analyses, raw data, and numbers of replicates for all analyses.

### 4.4 Chemotaxis assay

To access the preference for vitamin A, VA was applied to only one side of the bacterial lawn, the plates were allowed to dry in the incubator for hours. A drop of M9 buffer containing 100–200 age‐synchronized Day 1 adult worms were placed in the center of the chemotaxis plate and allowed to move freely at 15°C. The amount of worms in either areas were scored after 1 h to calculate the chemotaxis index (CI) using the following formula: CI = (A − B)/(A + B) (Margie et al., 2013). A and B represent the number of worms at VA and control positions. The experiments were independently repeated at least three times.

### 4.5 UPLC‐MS/MS analysis

To check concentration of VA in *C. elegans*, age‐synchronized Day 1 adult worms were pretreated with or without 5 mg/mL of this vitamin for 5 days (600–800 worms per condition). The major compounds within *C. elegans* were measured and quantified by liquid chromatography coupled with mass spectrometry (MS). Worm pellet (~0.1 mL for each sample) was mixed with methanol: acetonitrile (4:6, vol:vol) solution, then MS grade n‐hexane (Sigma‐Aldrich) was added during vortex extraction. Samples were analyzed by ultra‐high performance liquid chromatography (UPLC, Waters ACQUITY UPLC) and MS (AB Sciex 4000) using a 5‐μm TSK GEL‐ODS100V (150 × 2.1 mm) column (Tosoh Biosciences) at 0.4 mL/min and 40°C with the vol:vol ratio of 0.1% acetic acid as the aqueous mobile phase and acetonitrile as the organic phase 3:7 (the experiments were independently repeated at least three times).

### 4.6 Oxidative stress resistance assay

To test the effect of vitamin A on the survival and ROS accumulation in *C. elegans* challenged by juglone‐induced oxidative stress, synchronized wild‐type N2, *skn‐1(zu67)*, or *pmk‐1(km25)* worms were grown on NGM plates until they reached L4 stage and were transferred to 25°C. On the next day, synchronized Day 1 adult‐stage worms were moved to the NGM plates coated with 240 μM juglone (Yuanye Bio‐Tech) and 5 mg/mL VA. Worms were scored as dead or as alive everyday by tapping with a platinum wire. To measure the production of ROS, Day 1 adult worms were cultured on 240 μM juglone plate with or without 5 mg/mL VA for 3 days followed by ROS detection as described above (the experiments were independently repeated at least three times).

### 4.7 Cell culture and treatment

Human colorectal adenocarcinoma (Caco2) cells, a well‐established inflammation model (Greten & Grivennikov, 2019), and human embryonic lung fibroblast (IMR‐90) cells, which exhibit senescence‐associated phenotype in response to challenges, such as DNA damage caused by a chemotherapeutic drug doxorubicin (Baar et al., 2017) (Procell Life Science & Technology Co., Ltd., CL‐0050 and CL‐0538, respectively), were cultured with Dulbecco's Modified Eagle Medium containing 4.5 g/L D‐Glucose, L‐Glutamine, 110 mg/L Sodium Pyruvate (Gibco, C11995500) supplemented with 100 units/mL penicillin/streptomycin (Thermo Fisher), and 10% fetal bovine serum (Hyclone) at 37°C with 5% CO_2_. 27.9 μM VA was added to the medium after cells reached 70%–80% confluence and incubated for 48 h. To test doxorubicin (Aladdin)‐induced senescence in IMR90 cells, 48 h treatment with 27.9 μM VA was followed by incubation with 1 μM doxorubicin for 24 h (the experiments were independently repeated at least three times).

### 4.8 Quantitative real‐time PCR analysis

Total RNA from *C. elegans*, cells, and mouse liver tissues was extracted by Trizol using RNAiso plus reagent (Takara bio). Reverse transcription by PrimeScript RT Reagent kit with gDNA Eraser (Takara RR047A) was followed by qRT‐PCR in CFX96 Real‐Time PCR (Bio‐Rad) using TB Green Premix Ex Taq II kit (Takara bio). Fold changes in mRNA expression were calculated by △△Ct method using *C. elegans gpd‐1*, human *GAPDH*, and mouse *Gapdh* as reference genes. The sequences of the primers are listed in Table S6.

### 4.9 Statistical analysis

Mean survival in the primary screening was analyzed by the Mann–Whitney test with false discovery rate (FDR) correction. Survival was analyzed by the log‐rank (Mantel–cox) test, mean lifespan—by OASIS2 (Han et al., 2016) and compared by one‐way ANOVA followed by Tukey's post hoc test, the means between two groups—by Student's unpaired *t* test in GraphPad Prism 9. *p* Values <0.05 were considered statistically significant. All data are expressed as mean value ± standard error of means (SEM). Biological replicates were used in all experiments (*n* for each group in experiments is indicated in the figures or in the respective legends).

## AUTHOR CONTRIBUTIONS

Conceptualization: C.S., J.W., and I.A.V. Data curation: C.S., D.L., Z.Z, J.W., and I.A.V. Formal analysis: C.S., D.L., Z.Z, J.W., and I.A.V. Funding acquisition: A.P.S., Y.L., J.W., and I.A.V. Investigation: C.S., D.L., X.W., Z.Z,, R.K., S.H., W.L., Y.L., and J.W. Methodology: C.S., A.P.S., J.W., and I.A.V. Project administration: C.S., A.P.S., J.W., and I.A.V. Resources: C.S., A.P.S., J.W., and I.A.V. Software: C.S. and D.L. Supervision: C.S., A.P.S., J.W., and I.A.V. Validation: C.S., D.L., Z.Z, J.W., and I.A.V. Visualization: C.S., D.L., and I.A.V. Writing the original draft: C.S. and I.A.V. Writing the review and editing: C.S., J.W., and I.A.V. All authors read and approved the final version of the manuscript.

## FUNDING INFORMATION

This study was funded by the National Natural Science Foundation of China grants BC0800399 and BC0800441 to IAV and the joint seed fund of the Shanghai Jiao Tong University‐University of Warwick grant WH610160507 to IAV and APS.

## CONFLICT OF INTEREST STATEMENT

None declared.

## Supporting information


Appendix S1.

Tables S1–S5.

## Data Availability

All data are included in the manuscript.
