# Supplementary material for: SKN‐1/NRF2 upregulation by vitamin A is conserved from nematodes to mammals and is critical for lifespan extension in Caenorhabditis elegans
Source: Aging Cell. 2023 Dec 15;23(3):e14064. doi: 10.1111/acel.14064 (PMC10928581; doi:10.1111/acel.14064)
Supplement: Supplementary file 1 — Appendix S1. [file ACEL-23-e14064-s002.pdf]

Supplementary information for

SKN-1/NRF2 up-regulation by vitamin A is conserved from nematodes to mammals and is critical for lifespan extension in *Caenorhabditis elegans*

Chaweewan Sirakawin[1], Dongfa Lin[1,2], Ziyue Zhou[1], Xiaoxin Wang[3], Rhianne Kelleher[4], Shangyuan Huang[1], Weimiao Long[1], Andre Pires-daSilva[4], Yu Liu[1],

Jingjing Wang[3,✉], Ilya A. Vinnikov[1,✉]

This file contains:
Figure S1
Figure legends for Tables S1-S5
Table S6

Tables S1-S5 can be found in the separate file **Tables S1-S5.xlsx**

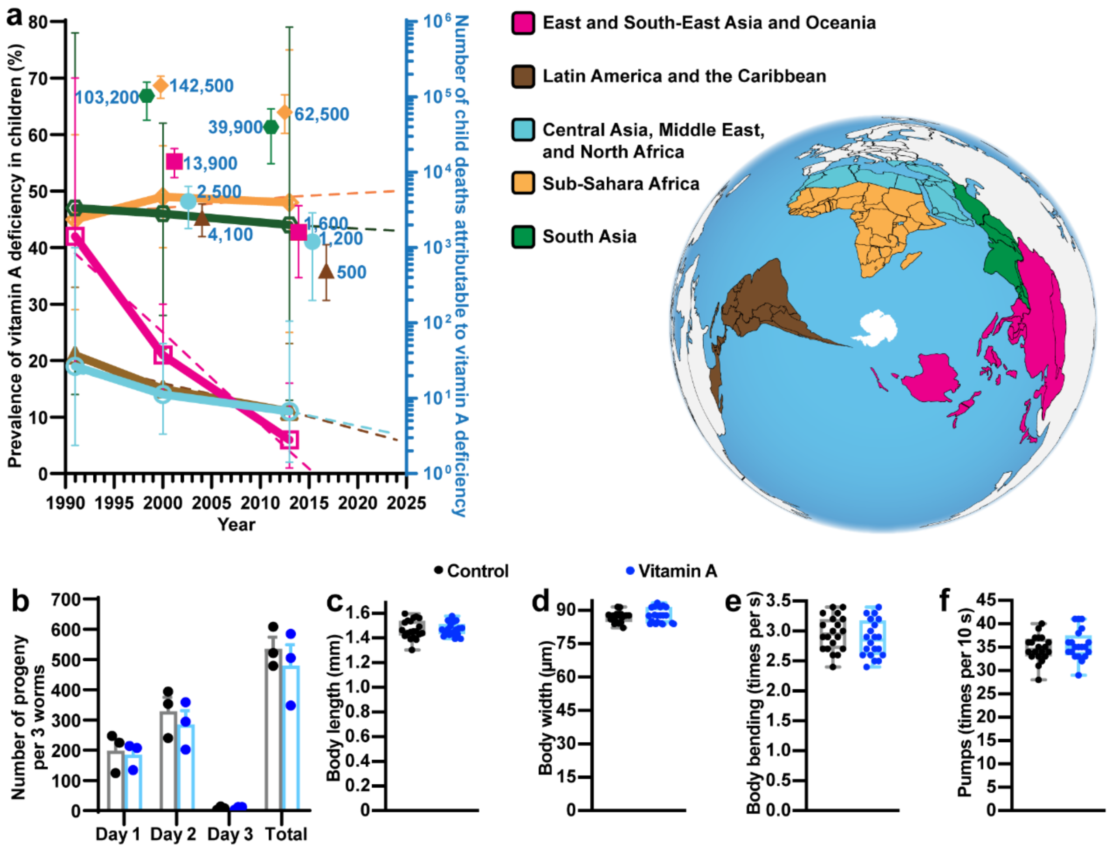

Fig. S1. Epidemiological studies on vitamin A deficiency and phenotypic analyses in vitamin A-treated *C. elegans*. a) Prevalence of vitamin A deficiency in children and child deaths attributed to it in southern parts of the world, based on Stevens et al. (Stevens et al., 2015). **b-f**) Fecundity assessed on days 1-3 of adulthood (**b**), body length (**c**), body width (**d**) body bending (**e**) and pharyngeal pumping (**f**) assessed on day 5 of adulthood in *C. elegans* treated with 5 mg/mL VA (n=3). Data are presented as means ± SEM.

Tables S1-S5 can be found in the separate file **Tables S1-S5.xlsx**

Table S1. List of chemicals and micronutrients used in the study.
Table S2. Analysis of primary survival screening in a 96 well format.
Table S3. Raw data of the primary survival screening in a 96 well format.
Table S4. Analysis of high confidence survival screening in a 6 cm dish format.
Table S5. Raw data of high confidence survival screening in a 6 cm dish format.

Table S6. Primer sequences used in quantitative real-time PCR analysis.

Genes	Primer Sequences (5'to 3')
gpd-1 **(*C. elegans*)**	F:GGCTTCTGCTCATCTTCAAGG<br>R:TGGTGCACGATGCGTTAGAA
pmk-1 **(*C. elegans*)**	F:TGCCCGTCGTACATATCGTG<br>R:GGTCATCGTTGAGTCGCTGA
skn-1 **(*C. elegans*)**	F:TTGGCGTGATGATCAACGGA<br>R:ACCGAATGGAGATGCTGGTG
gst-2 **(*C. elegans*)**	F:TGATGTGTTTCGATGTTCGAGG<br>R:CCGATTGAAGCCGACTGTGA
gst-3 **(*C. elegans*)**	F:TTTAATGCACGCGGACTTGC<br>R:TGAGCTCCGTCTATGCACAA
gst-4 **(*C. elegans*)**	F:TGCTCTTGCTGAGCCAATCCGT<br>R:CCAGCGAGTCCAAATTTTCTTGCCA
gst-5 **(*C. elegans*)**	F:CTACTTCAATGGACGTGGCG<br>R:ATGCTGGCCATTGTTCCTGA
gst-6 **(*C. elegans*)**	F:CCATTAAGAGCCCGTGCTGA<br>R:ATTTTTGCGTGCTGGCCATT
gst-11 **(*C. elegans*)**	F:GGCTTATCTTCGCCTACGCA<br>R:TCTGTTTCTCCGGCAAGTCC
gst-12 **(*C. elegans*)**	F:ATCTCCGCGCAAAGTGCATA<br>R:AAACGGAACTCCGGCAAGAT
gst-16 **(*C. elegans*)**	F:CTCCCGAAGAAGAAGCCTGG<br>R:CAGCAGATTTTCCACTGCGT
gst-20 **(*C. elegans*)**	F:CGGACGAGGATTGGGAGATG<br>R:TTTGGAGTCCCGAACTGAGC
gst-22 **(*C. elegans*)**	F:GCCGACGATCAATGCTGTTC<br>R:CTTCCGGTGTTTTTCCAGCG
gst-33 **(*C. elegans*)**	F:TTTGCTGGACAAACTCCGGT<br>R:CTCCTTAGCAGGGTCCAACG
GAPDH **(Human)**	F:GGAGCGAGATCCCTCCAAAAT<br>R:GGCTGTTGTCATACTTCTCATGG
CCL2 **(Human)**	F:AAGACCATTGTGGCCAAGGA<br>R:TTCGGAGTTTGGGTTTGCT
MMP-1 **(Human)**	F:AGCCTTCCAACTCTGGAGTAATGT<br>R:CCGATGATCTCCCCTGACAA
IL-6 **(Human)**	F:ACTCACCTCTTCAGAACGAATTG<br>R:CCATCTTTGGAAGGTTCAGGTTG
IFN-β **(Human)**	F:AAACTCATGAGCAGTCTGCA<br>R:AGGAGATCTTCAGTTTCGGAGG
NRF2 **(Human)**	F:TCCAGTCAGAAACCAGTGGAT<br>R:GAATGTCTGCGCCAAAAGCTG
p21 **(Mouse)**	F:CAGATCCACAGCGATATCCA<br>R:ACGGGACCGAAGAGACAAC
Gapdh **(Mouse)**	F:FAGGTCGGTGTGAACGGATTTG<br>R:RTGTAGACCATGTAGTTGAGGTCA
